# P-1995. A Nationwide Cohort Study of Delta and Omicron SARS-CoV-2 Outcomes and Vaccine Effectiveness amongst Individuals with Chronic Heart Failure or Ischemic Heart Disease

**DOI:** 10.1093/ofid/ofae631.2153

**Published:** 2025-01-29

**Authors:** Liang En Wee, Enoch Xueheng Loy, Jue Tao Lim, Sharon Tan, David Lye, Kelvin Bryan Tan

**Affiliations:** Singapore General Hospital, Singapore, Not Applicable, Singapore; National Centre for Infectious Diseases, Singapore, Not Applicable, Singapore; LKCMedicine, NTU, Singapore, Singapore; National Centre for Infectious Diseases, Singapore, Not Applicable, Singapore; National Centre for Infectious Diseases, Singapore, Not Applicable, Singapore; National Centre for Infectious Diseases / Ministry of Health, Singapore, Not Applicable, Singapore

## Abstract

**Background:**

Individuals with heart failure (HF) / ischemic heart disease (IHD) have poorer outcomes following respiratory viral infections; however, data is lacking on outcomes and Vaccine Effectiveness (VEs) following SARS-CoV-2 infection with newer COVID variants.

**Methods:**

Outcomes of Delta/Omicron SARS-CoV-2 infection in a highly vaccinated/boosted cohort of adult Singaporeans with HF/IHD were contrasted against matched population controls. Matching was conducted based on age, sex, ethnicity, socioeconomic status, and comorbidities. Cox regressions were used to compare risk of infection, COVID-19-related hospitalisations and severe COVID-19 disease and estimate VEs.

**Results:**

15,426 HF patients and 110,442 IHD patients were matched to 41,221 and 223,843 population controls, respectively. Compared with controls, higher risk of COVID-19 hospitalisation and severe disease was observed amongst HF cases during Delta (hospitalisation: adjusted-hazards-ratio, aHR=1.40[1.20-1.62]; severe-COVID-19: aHR=1.66[1.28-2.14]) and Omicron (hospitalisation: aHR=1.77[1.65-1.90]; severe-COVID-19: aHR=2.40[2.10-2.75]). Higher risks were also observed for infected IHD patients, albeit to a smaller extent (Omicron, hospitalization: aHR=1.21[1.17-1.26]; severe-COVID-19: aHR=1.12[1.03-1.21]). During both Delta and Omicron waves, the risk of COVID-19-related infection, hospitalisation and severe disease was significantly lower in boosted groups compared against those who had only received 2 doses. VEs were comparable with controls for IHD, with lower effectiveness observed against hospitalisation amongst HF patients (3 doses aHR=0.42[0.37-0.48]; 4 doses aHR=0.28[0.23-0.34]) compared to controls (3 doses: aHR=0.28[0.23-0.34]; 4 doses: aHR=0.18[0.16-0.22]).

**Conclusion:**

Higher risks of severe COVID and comparable VEs suggest that it is more cost effective to boost IHD/HF patients. Lower VEs amongst HF patients point to the need for more boosters to achieve similar rates of protection.

**Disclosures:**

All Authors: No reported disclosures

